# Deoxynivalenol Induces Inflammation in the Small Intestine of Weaned Rabbits by Activating Mitogen-Activated Protein Kinase Signaling

**DOI:** 10.3389/fvets.2021.632599

**Published:** 2021-02-02

**Authors:** Pengwei Wang, Libo Huang, Wanying Yang, Quancheng Liu, Fuchang Li, Chunyang Wang

**Affiliations:** Shandong Provincial Key Laboratory of Animal Biotechnology and Disease Control and Prevention, Shandong Agricultural University, Taian, China

**Keywords:** deoxynivalenol, intestinal inflammation, weaned rabbit, ERK-p38 signal pathway, PKR, Hck

## Abstract

Deoxynivalenol (DON) can activate related signaling pathways and induce gastrointestinal disorders. Based on the results of previous studies, this study tried to explore the relationship between DON-induced intestinal inflammation of weaned rabbits and the ERK-p38 signaling pathway. Forty-five weaned rabbits were divided into three treatments: control, LD and HD group. All rabbits were treated with diet containing a same nutrient content, but animals in the LD and HD groups were additionally administered DON via drinking water at 0.5 and 1.5 mg/kg b.w./d, respectively. The protocol consisted of a total feeding period of 31 days, including a pre-feeding period of 7 days. Western blotting, qRT-PCR, and immunohistochemistry were applied for analysis the expression of protein and mRNA of extracellular signal-regulated kinase (ERK), p38, double-stranded RNA-activated protein kinase (PKR), and hematopoietic cell kinase (Hck) in the duodenum, jejunum, and ileum of rabbits, as well as the distribution of positive reactants. The results proved that DON intake could enhance the levels of inflammatory factors in serum and damage the intestinal structure barrier of rabbits. Meanwhile, DON addition can stimulate the protein and mRNA expression for ERK, p38, PKR, and Hck in the intestine of rabbits, especially in the duodenum, as well as expand the distribution of positive reactants, in a dose-dependent manner.

## Introduction

Contamination of agricultural products by natural mycotoxins has been a long-standing challenge for agriculture and food industries. Deoxynivalenol (DON), which is produced by fungi, is classified as a type B trichothecene (1). DON is also called vomitoxin because it can cause vomiting in pigs (2). In view of its contamination rate and toxic effects, DON is considered to be one of the most common mycotoxins in grain products and feed ingredient worldwide (3, 4). DON is chemically stable and has a variety of toxic effects, such as genotoxicity, cytotoxicity, reproductive toxicity, neurotoxicity, and immunotoxicity (5, 6). Therefore, it can seriously threaten human and animal health by contaminating food and feed.

The small intestine is the first physical barrier against the entry of DON and other mycotoxins into the body (7). Numerous studies have concluded that DON intake can induce intestinal lesions in porcine and poultry models (8–10). Porcine species are generally considered the most sensitive animals to DON. Most ingested DON in contaminated feeds is rapidly absorbed in the porcine small intestinal segment (11), causing morphological and histological damage in the small intestine, inhibiting the absorption of nutrients by the intestine, and resulting in appetite suppression and decreased growth performance (12). The latest findings concerning the underlying molecular toxicological mechanism of DON focused on intracellular signaling pathways (13, 14). Therefore, it is necessary to further explore the potential molecular mechanism of intestinal inflammation induced by DON through *in vivo* experiments.

Mitogen-activated protein kinases (MAPKs), including p38 and extracellular signal-regulated kinase (ERK), comprise a family of signaling proteins (15), and they are crucial to the pro-inflammatory gene expression and apoptosis induced by DON (16–18). Activation of ERK plays a pivotal role in inducing cell motility, proliferation, differentiation, and survival, whereas p38 is closely related to cell cycle progression and cell differentiation (19). After ERK and p38 are activated, they phosphorylate MAPK, promote the expression of immune genes, and cause intestinal function damage (20). The activation of MAPKs induced by DON involves two key upstream signal transductive factors, namely double-stranded RNA-activated protein kinase (PKR) and hematopoietic cell kinase (Hck) (21). PKR and Hck can mediate the DON-induced activation of ERK and p38, thereby inducing apoptosis, inflammatory factor expression, and immunosuppressive effects (22). The participation of PKR and Hck is essential to p38 ribosome recruitment induced by DON and protein phosphorylation, and they activate the expression of pro-inflammatory cytokines driven by p38 (23).

The rabbit is an important model animal for the study of human diseases and nutrition, as well as a high-quality source of meat. With the wide application of full-price feed in the rabbit industry, issues concerning contamination by mycotoxins in feed and potential threats to food safety have attracted increasing attention from scientists (24, 25). Rabbits are monogastric herbivores, and their intestinal structure is quite different from that of pigs and poultry (26). However, few studies have assessed the toxic effects and mechanism of DON in rabbits, especially concerning the correlation between MAPK signaling and intestinal inflammation induced by DON. Therefore, this research aimed to analysis the variation tendency of ERK-p38 signaling pathway and upstream signaling factors PKR and Hck in the various intestine segments of weaned rabbits induce by DON.

## Materials and Methods

### Ethics Statement

The operation procedures concerning live animals in this paper complied with the ethical regulations and the experimental protocol has been ratified by the Protection Committee of Shandong Agricultural University.

### DON Source

DON (C_15_H_20_O_6_), supplied by Triplebond Company (Guelph, Ontario, Canada), were diluted to 1.5 and 0.5 mg/mL using sterile ultrapure water for later use.

### Experimental Design

The complete feed of weaned rabbits were purchased from Liuhe Feed Company (Taian, Shandong, China). All feeds were prepared a week before the experiment, and the composition of the experimental feeds and nutritional level determined by the normal method are listed in Table 1. Forty-five healthy 35-day-old weaned Rex rabbits were equally divided into three groups (mean weight, 879 ± 17.62 g), namely the control, LD, and HD groups. Three replicates for each experimental group, and five rabbits per replicate. All rabbits were fed with basal diet, and animals in the LD and HD groups were additionally administered DON at 0.5 and 1.5 mg/kg b.w./d, respectively, via the drinking water. DON supplementation of the drinking water was performed as described by Yang et al. (27). The research spanned 31 days, including a 7-day pre-feeding period. On the morning of the 25th day of the experiment, a total of 36 rabbits on an empty stomach in three groups were selected for blood collection. After the blood drawn via the ear vein and centrifuged at 1,500 × g for 15 min, the serum samples were separated and stored at −20°C. On the same day, 18 rabbits (six per treatment) were euthanized by injection, and then intestinal samples were quickly obtained from the mid-segment of the duodenum, jejunum, and ileum and stored at −70°C for later use. Additionally, some of the intestinal samples were fixed using Bouin's solution and washed with 0.9% NaCl solution immediately, and the remaining samples were stored at −70° for later analysis.

**Table 1:** The ingredient and composition of the basic feed.

**Ingredient (%)**		**Calculated composition**	
Maize	14	Dry matter	88.64
Soybean meal	17	Crude protein	20.05
Wheat bran	13	Crude fiber	18.78
Corn germ meal	19	Crude ash	10.45
Rice hulls	10	Crude fat	3.34
Soybean straw powder	7	Calcium	0.72
Alfalfa	10	Total phosphorus	0.55
Malt sprout	5	Digestible energy (MJ/kg)	10.06
Sweet wormwood	3.5		
Premix material^a^	1.5		
Total	100		

a *Premix material provided per kilogram of feed: VA, 12,000 IU; Total VB, 63.52 mg; choline, 600 mg; biotin, 0.2 mg; VD 3,100 IU; VE, 50 mg; VK, 31.5 mg; Fe, 60 mg; Zn, 60 mg; Cu, 40 mg; Mn, 9 mg; Se, 0.2 mg; NaCl, 5,000 mg; lysine, 1,500 mg; and methionine, 1,000 mg*.

### Enzyme-Linked Immunosorbent Assay (ELISA)

Conventional ELISA method was used to detect the content of several inflammatory factors in serum samples of rabbits, including tumor necrosis factor-α (TNF-α), prostaglandin E synthase 2 (PTGES2), interleukin-1β (IL-1β), interleukin-2 (IL-2), interleukin-6 (IL-6), and interleukin-8 (IL-8). The operating procedure strictly followed the instructions of the ELISA kit, which was purchased from Quanzhou Kenuodi Biology Co., Ltd. of China. The optical density (OD) of the tested standard was determined as the abscissa, and the concentration value of the standard was determined as the ordinate after the experiment. Then, the standard curve was constructed using Excel, and the linear regression equation was obtained. The OD values of serum samples obtained at 450 nm using a microplate reader were substituted into the equation, and then the concentrations of samples were calculated.

### Western Blotting

The total protein of the small intestinal samples was extracted via Protein Extraction Kit (Beyotime, Shanghai, China), then conventional BCA was used to measure total protein concentrations. A PAGE gel was prepared using a PAGE Gel Fast Preparation Kit (EpiZyme, Shanghai, China). After sample addition, electrophoresis was performed using PowerPac Basic (Bio-Rad, Singapore) at 80–110 V. Then, the Immobilon-P transfer membrane (Merck Millipore, Boston, US) was activated in methanol, and proteins were transferred at 200 mA for 2 h. Western Blocking Buffer (Beyotime) was used to block non-specific binding. The membrane was then incubated with primary specific polyclonal rabbit antibodies against ERK1/2, p38, PKR, and Hck (1:2,000; BIOSS, Beijing, China) or β-actin (1:300; Beyotime) at 4°C overnight. After washed using TBS-Tween three times, the membrane was subsequently incubated with secondary antibodies at 4°C for 2 h. A BeyoECL Plus P0081 88 kit were used to color the bands. After captured by FusionCapt Advance FX7 (Fusion FX; OSTC Ltd. San Diego, CA, USA), the images were processed using Image Pro-Plus 6.0 software (Media Cybernetics, Silver Spring, MD, USA) to acquire quantitative data following the steps published by Wu et al. (28).

### Quantitative Reverse Transcription Polymerase Chain Reaction (qRT-PCR)

After the total RNA were extracted by TRIzol (Invitrogen Co., Carlsbad, CA, USA), the concentration and quality of RNA taken from intestine samples of rabbits were confirmed spectrophotometrically. RNA was then immediately reverse-transcribed following the instructions of the PrimeScript@ RT Master Mix Perfect Real-Time kit (Perfect Real Time; TaKaRa, Shiga, Japan). Primer sequences for p38, ERK, PKR, Hck, and GAPDH, obtained from NCBI, are listed in Table 2. The total reaction volume in this experiment was 20 μL, including 10 μL of SYBR Premix Ex Taq, 0.4 μL of forward primers (10 μM/L), 0.4 μL of reverse primers (10 μM/L), 0.4 μL of ROX reference dye, 2 μL of cDNA, and 6.8 μL of dH_2_O. The reaction procedure for real-time PCR was as follows: 15 s at 95°C and 40 cycles at 95°C for 15 s and 60 s at 60°C. The expression level of relative mRNA was computed using the 2^−ΔΔ*CT*^ method (29).

**Table 2:** Primer sequences of related genes used for qRT-PCR.

**Gene**	**Primer sequence(5′-3′)**	**GenBank no**.
*GAPDH-F*	*TGCCACCCACTCCTCTACCTTCG*	NM_001082253
*GAPDH-R*	*CCGGTGGTTTGAGGGCTCTTACT*	
*p38-F*	*GCTGATATTGGCGAGGATCTGGAC*	NC_000083.6
*P38-R*	*GCACGGCTCTGCTCACACTC*	
*ERK1/2-F*	*CGATGGTACAGAGCTCCCGA*	NC_009068.1
*ERK1/2-R*	*CAAGAGAGGCGTGGCTTGTT*	
*PKR-F*	*AGAACCAATTGGCGCAGGTG*	NC_035092.1
*PKR-R*	*ATCATGCCCGACCCAACAAC*	
*Hck-F*	*AGCCTGCTGGACTTCCTGAAGAG*	NC_013672.1
*Hck-R*	*CTCGGAGGTCTCGGTGGATGTAG*	

*ERK, extracellular signal-related kinase; PKR, double-stranded RNA-activated protein kinase; Hck, hematopoietic cell kinase; qRT-PCR, quantitative reverse transcription polymerase chain reaction*.

### Immunohistochemistry (IHC)

Routine IHC was performed using the two-step method with reference to the procedure published by Zhou et al. (30). The small intestinal samples of rabbits were cut into slices of ~5 μm thick, embedded in paraffin, attached to a glass slide coated with poly-l-lysine. Citric acid buffer (0.01 M, pH 6.0) was used for thermal unmasking of the antigen after dewaxing and debenzenizing. In order to block endogenous peroxidase, the slices were incubated using 3% H_2_O_2_. And then the DAB kit with primary polyclonal rabbit antibodies against ERK1/2, p38, PKP, and Hck (1:100; BIOSS) were used for immunohistochemical staining. The secondary antibody was Polink-2 plus immunohistochemical assay kit (ZSBIO, Beijing, China), and 10% calf serum was used for the seal. The slices were stained with hematoxylin, dehydrated, cleared, and sealed with transparent resin. Finally, immunopositive cells with brownish yellow color could be observed under a microscope. Two indicators, namely the total cross-sectional integrated optical density (IOD) and light density of single intestinal villi (SIOD), were calculated using Image Pro-Plus 6.0 software (Media Cybernetics, Silver Spring, MD USA) based on cell staining and used to assess the intensity of positive reaction.

### Data Statistics

SPSS 21.0 for Windows was applied to analysis for all data, including the data from image scanning. In addition, one-way analysis of variance and Tukey's test were used to compare the average values among the groups. All results were expressed using the mean ± SD, and *p* < 0.05 was considered statistically significant. Western blotting data were expressed as the ratio of the gray value of p38, ERK, PKR, and Hck bands to the gray value of the internal reference band.

## Results

### Concentrations of Inflammatory Factors in Serum

**Figure 1:**
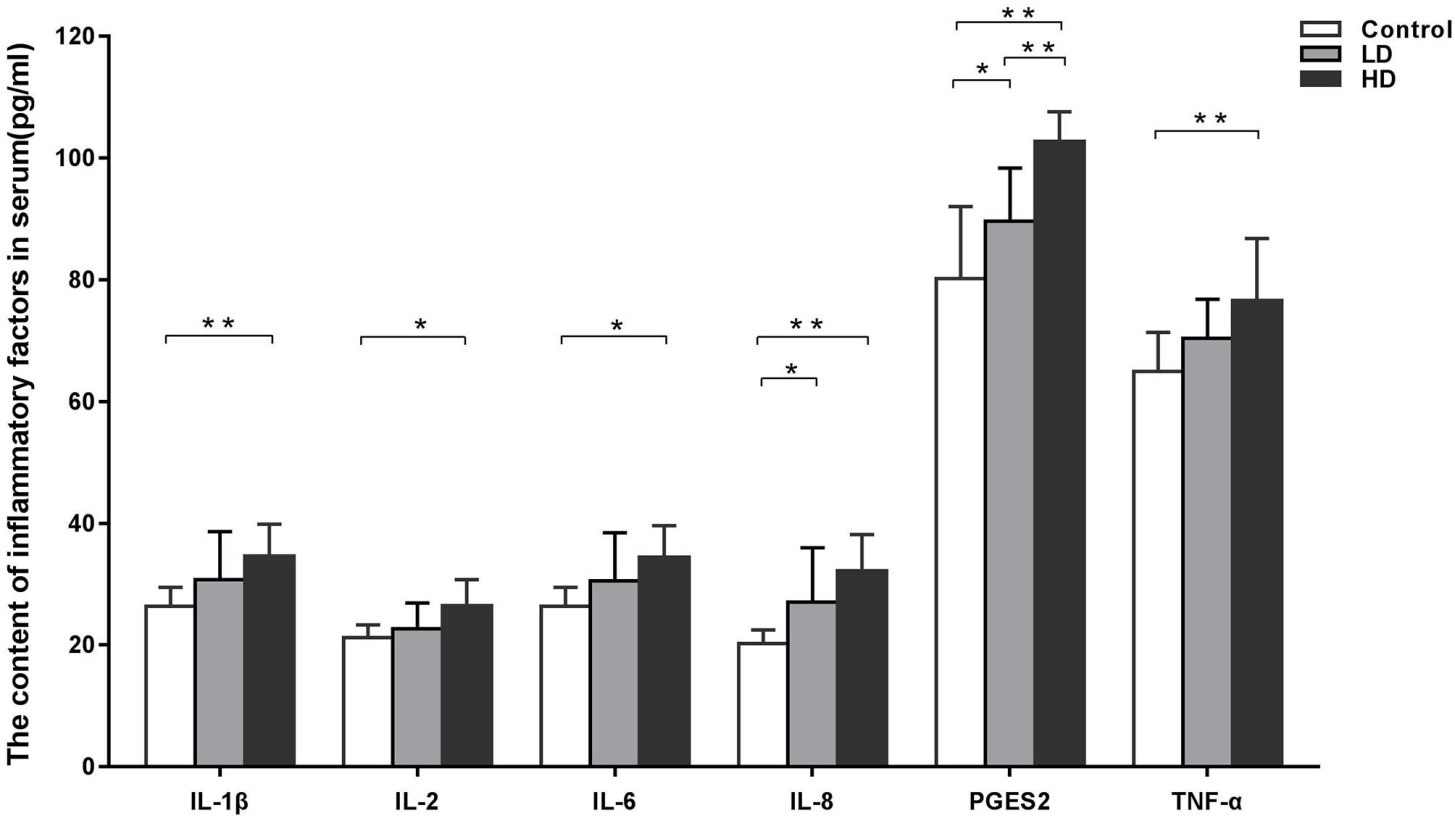
Inflammatory factors in serum of weaned rabbits among different treatment (M ± SD, *n* = 10). Control, LD, and HD refer to the different treatment. ^**^ and ^*^, respectively, means difference significant at *p* < 0.01 and *p* < 0.05.

The effects of DON supplementation on serum inflammatory factor levels in weaned rabbits are presented in Figure 1. Compared to the control group data, the levels of IL-1β, IL-2, IL-6, IL-8, TNF-α, and PTGES2 in the HD group were significantly increased (*p* < 0.05), while the levels of PTGES2 in the LD group were also enhanced obviously (*p* < 0.05).

### Expression and Distribution of ERK1/2

**Figure 2:**
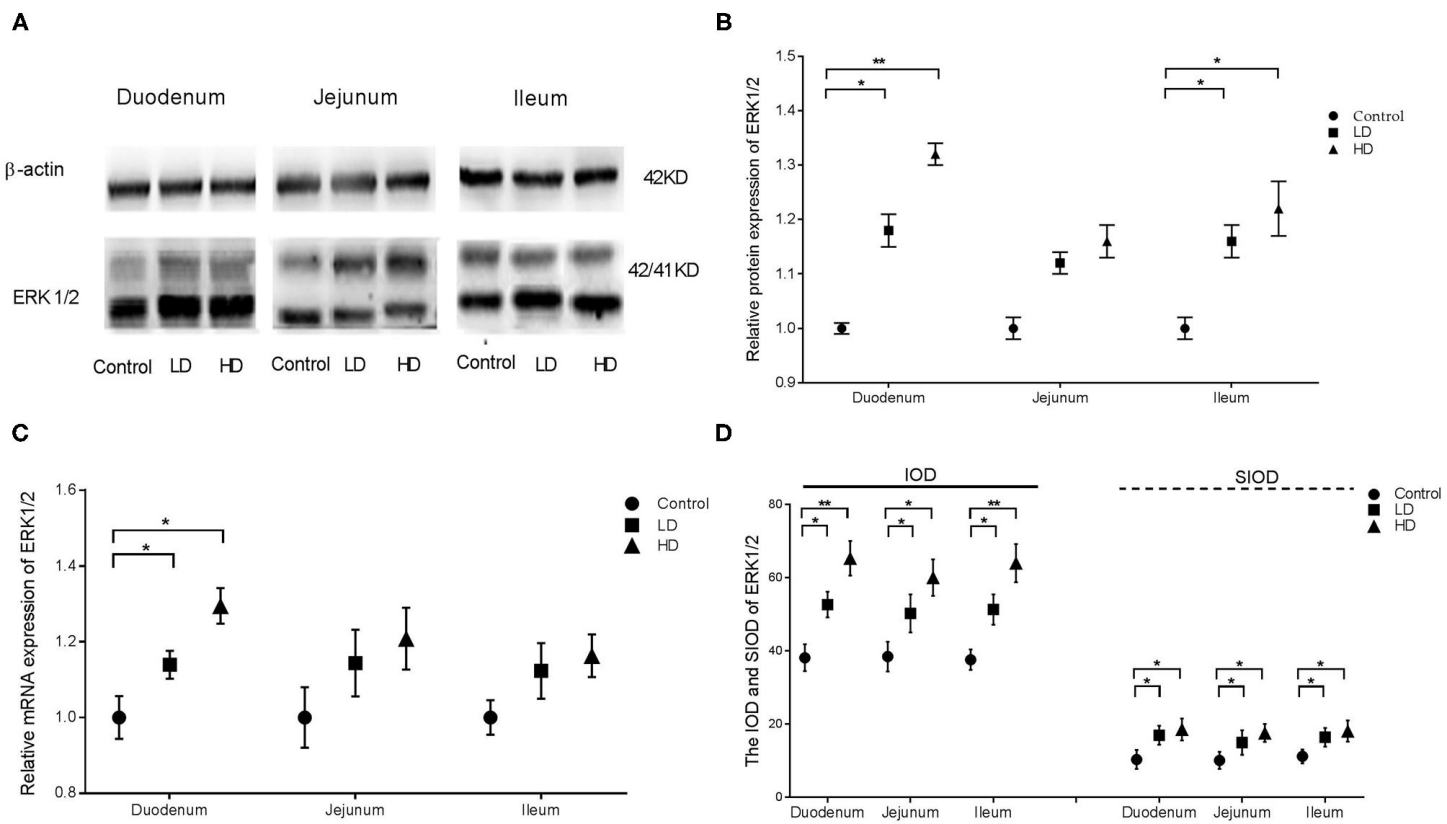
Distribution and expression of ERK1/2 in intestine induced by DON (M ± SD, *n* = 6). **(A)** Typical protein expression bands of ERK1/2. **(B)** Relative protein expression of ERK1/2. **(C)** Relative mRNA expression of ERK1/2. **(D)** IOD and SIOD of ERK1/2. Control, LD, and HD refer to the different treatment. ^**^ and ^*^, respectively, means difference significant at *p* < 0.01 and *p* < 0.05.

Compared with the control group findings, protein expression of ERK1/2 in the duodenum and ileum were significantly improved (*p* < 0.05) by DON treatment, but those in the jejunum was not changed (Figures 2A,B). Regardless of the low-dose or high-dose group of DON treatment, the mRNA expression of ERK1/2 were significantly more (*p* < 0.05) than that of the control group only in the duodenum (Figure 2C). The IOD and SIOD of ERK1/2 in the three intestinal segments were significantly higher (*p* < 0.05) both in two dose DON treatments, especially in the higher-dose group, than in the control group (Figure 2D). Photographs from immunohistochemical staining of various intestinal segments (Supplementary Figure 1) indicated that positivity for ERK1/2 were mainly distributed within villi in the control group, and a bit of positive material was also detected in the lamina propria. However, the levels of positive reactants distributed in the intestinal glands and villous epithelium were increased obviously after DON exposure, especially in the higher-dose group.

### Expression and Distribution of p38

**Figure 3:**
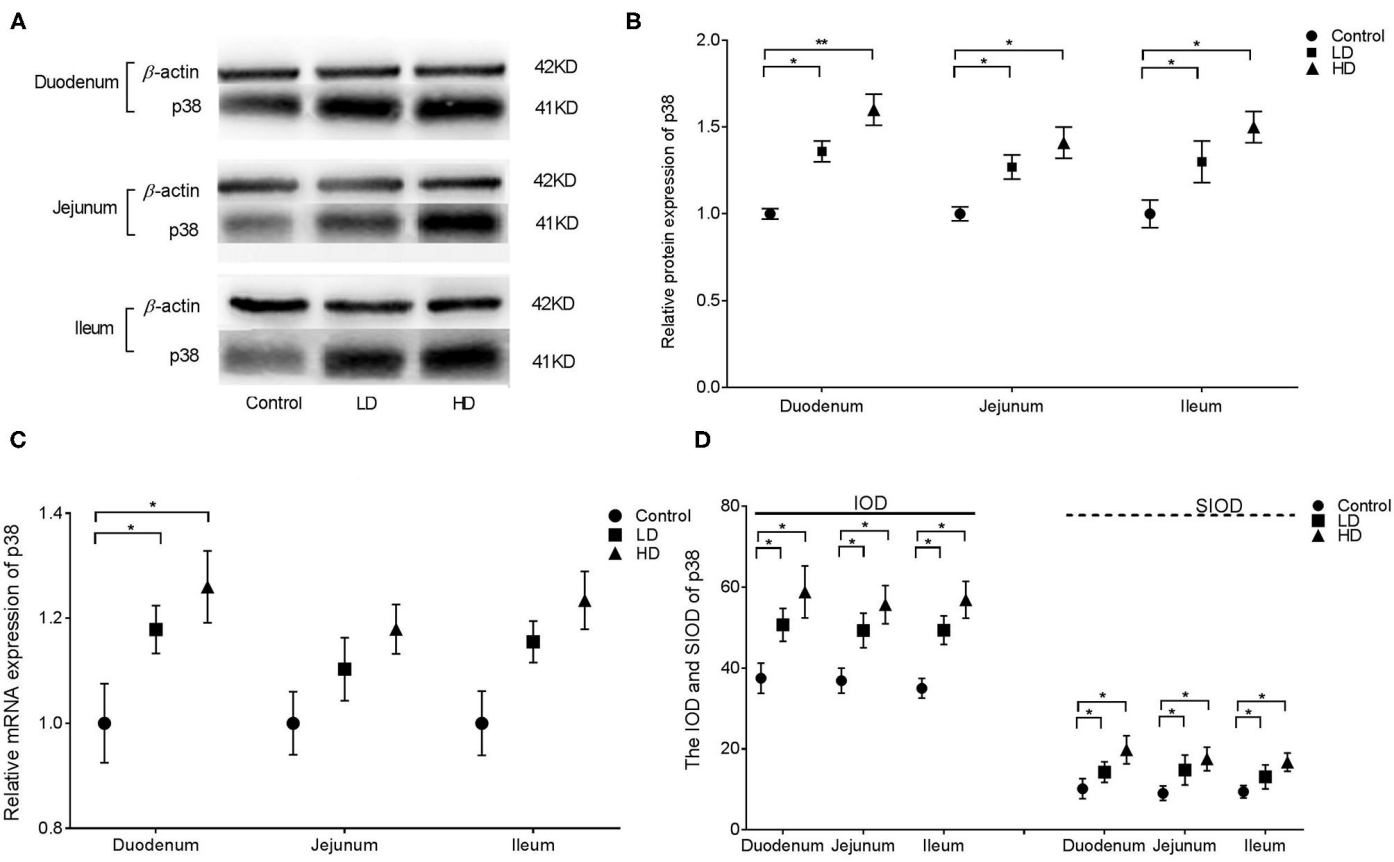
Distribution and expression of p38 in intestine by DON (M ± SD, *n* = 6). **(A)** Typical protein expression bands of p38. **(B)** Relative protein expression of p38. **(C)** Relative mRNA expression of p38. **(D)** IOD and SIOD of p38. Control, LD, and HD refer to the different treatment. ^**^ and ^*^, respectively, means difference significant at *p* < 0.01 and *p* < 0.05.

The results from western blotting revealed that p38 protein expression in various intestinal segments were significantly higher (*p* < 0.05) by DON treatment than in the control group (Figures 3A,B). Conversely, the mRNA expression of p38 in the duodenum was only significantly increased (*p* < 0.05) after DON treatment (Figure 3C). Regardless of the low-dose or high-dose group of DON treatment, the IOD and SIOD value of p38 in the various intestinal segments of rabbits were significantly more (*p* < 0.05) than in the control group (Figure 3D). As presented in Supplementary Figure 2, IHC revealed positivity for p38 in control rabbits primarily in the lamina propria of the intestine glands, whereas staining in the epithelium was less intense. However, after DON consumption, the distribution of positive reactants for p38 in all three intestinal segments were enlarged obviously compared to the control group findings, and positivity were mainly distributed in the villi epithelium and small intestine glands.

### Expression and Distribution of PKR

**Figure 4:**
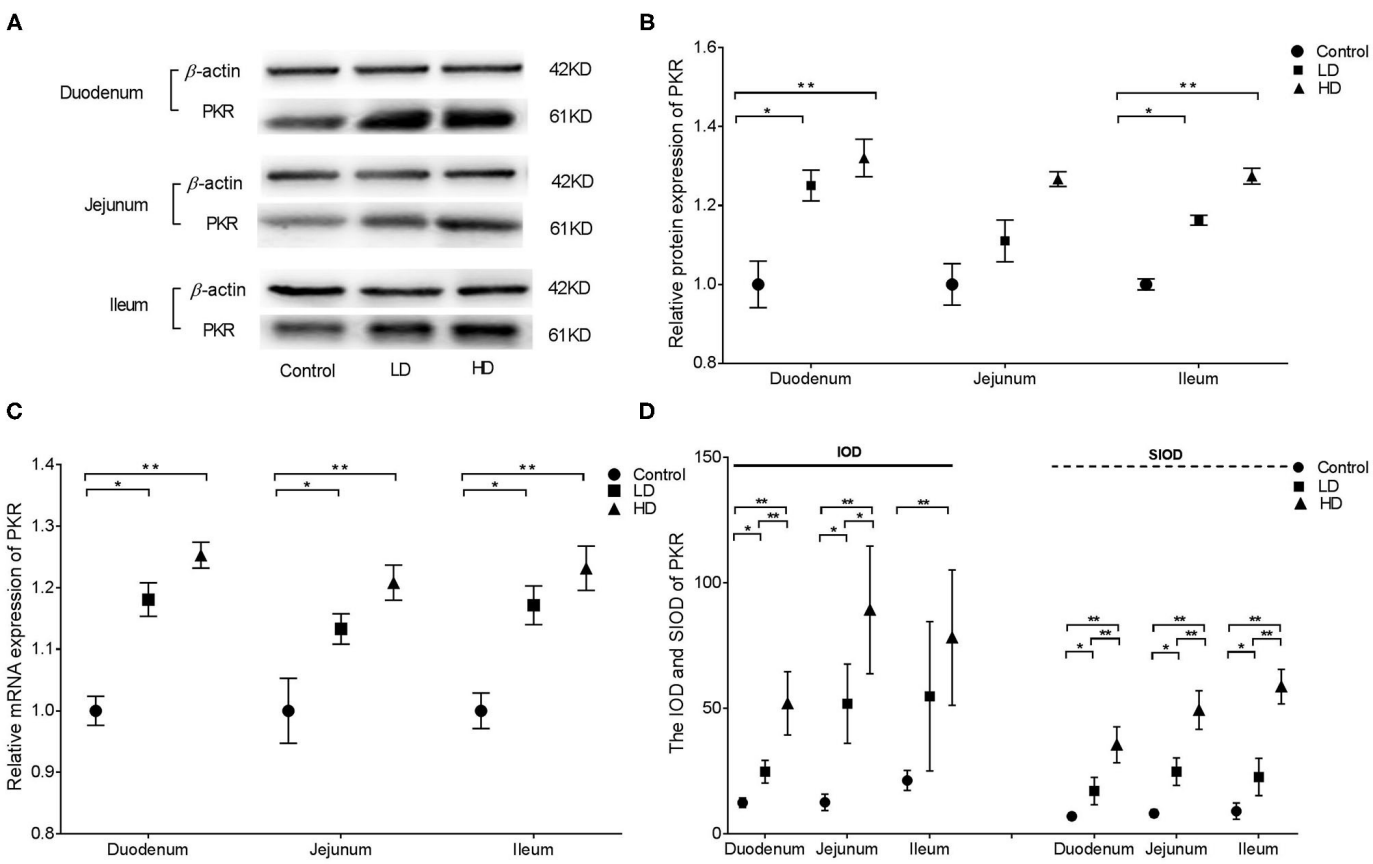
Distribution and expression of PKR in three intestine induced by DON (M ± SD, *n* = 6). **(A)** Typical protein expression bands of PKR. **(B)** Relative protein expression of PKR. **(C)** Relative mRNA expression of PKR. **(D)** IOD and SIOD of PKR. Control, LD, and HD refer to the different treatment. ^**^ and ^*^, respectively, means difference significant at *p* < 0.01 and *p* < 0.05.

Western blotting revealed that PKR protein expression in the duodenum and ileum of rabbits were significantly higher (*p* < 0.05) by DON treatment compared with the control value, whereas no difference was noted in the jejunum (Figures 4A,B). Regardless of the low-dose or high-dose group of DON treatment, the relative mRNA expression of PKR in the various intestinal segments of rabbits were significantly higher (*p* < 0.05) than in the control group (Figure 4C). Compared with the control group findings, the IOD value of PKR in duodenum and jejunum of rabbits was significantly higher (*p* < 0.05) after DON exposure, and a significant difference was noted between two dose DON treatment (*p* < 0.05). The IOD value of PKR in the ileum was significantly increased (*p* < 0.05) only in the HD group compared with the control value. The SIOD of PKR was significantly increased in all three intestinal segments (*p* < 0.05) after DON consumption, and the values were significantly higher in the HD group than in the LD group in all three segments (Figure 4D). IHC (Supplementary Figure 3) revealed that the positivity of PKR were mainly distributed in the intestine glands for control rabbits, whereas the distribution of positivity of PKR in the intestinal villi and glands were enlarged in two dose DON treatment. As the DON dose was increased, clumps of positively stained reactants were most evident in villous epithelial cells at the tips of the plicae villi in the duodenum and in the intestinal glands in the ileum.

### Expression and Distribution of Hck

**Figure 5:**
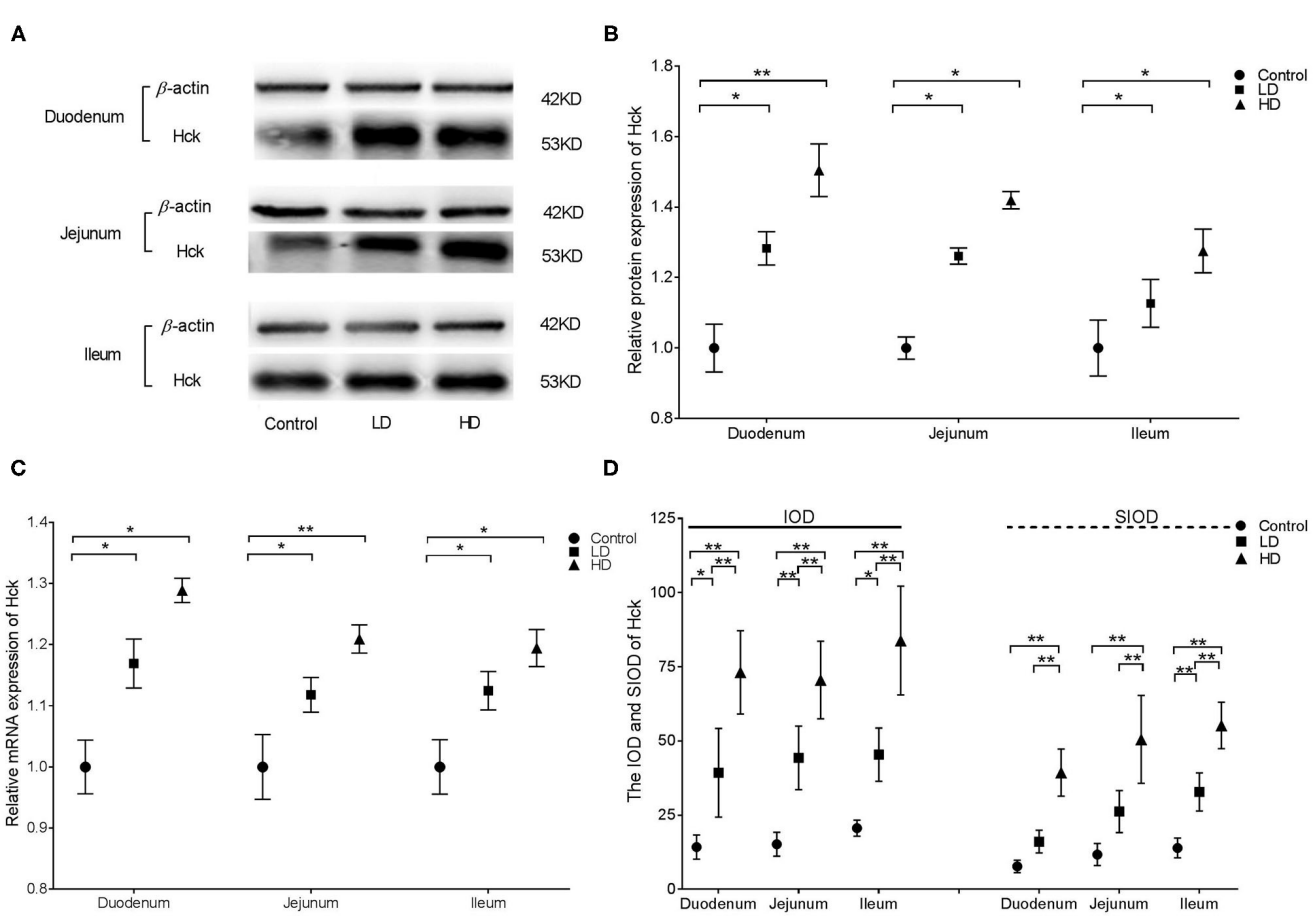
Distribution and expression of Hck in intestine induced by DON (M ± SD, *n* = 6). **(A)** Typical protein expression bands of Hck. **(B)** Relative protein expression of Hck. **(C)** Relative mRNA expression of Hck. **(D)** IOD and SIOD of Hck. Control, LD, and HD refer to the different treatment. ^**^ and ^*^, respectively, means difference significant at *p* < 0.01 and *p* < 0.05.

Western blotting illustrated that Hck protein expression in the various intestinal segments were significantly higher (*p* < 0.05) by DON treatment than in the control group (Figures 5A,B). qRT-PCR indicated that the relative mRNA expression of Hck in the duodenum, jejunum, and ileum of rabbits was significantly higher (*p* < 0.05) in both low-dose or high-dose group of DON treatment compared to the control value (Figure 5C). Regardless of the low-dose or high-dose group of DON treatment, the IOD data of Hck were significantly increased in all three intestinal segments (*p* < 0.05) compared with the data of control group, and significant differences were noted between two dose DON treatment (*p* < 0.05). The SIOD of Hck was significantly higher in all three intestinal segments (*p* < 0.05) in the HD group than in the control and LD groups, and the value in the duodenum was also significantly higher (*p* < 0.05) in the LD group than in the control group (Figure 5D). IHC (Supplementary Figure 4) revealed that the positivity for Hck were mainly distributed in the intestinal glands and villi epithelium in control rabbits, and the distribution range was obviously increased after DON exposure. As the DON dose was increased, clumps of positively stained reactants were most evident at the tips of the plicae and villi in duodenum and jejunum and in the intestinal grand in the ileum.

## Discussion

### Toxicity and Dose of DON

DON is considered to be a underlying health hazard for livestock farming and human health globally (31). Due to the different absorption pattern and metabolic pathways of different animal species, as well as composition of the intestinal flora, their sensitivity for DON is also different. Earlier researches have proved that pigs and rodents are the most sensitive to DON, and the minimum toxic dose were 0.03–0.12 mg/kg b.w. and 0.1–0.15 mg/kg b.w., respectively (32, 33). Pestka et al. have revealed that there were no decrease in growth performance or other signs of sickness even if the cows were treated using DON at 0.6 mg/kg b.w./d for 6 weeks or at 6.6 mg/kg b.w./d for 5 days (21). The relatively high tolerance of cattle for DON may be due to the fact that microorganisms in the rumen of cattle can reduce the toxic effect of DON by degrading DON into a series of non-toxic metabolites (1, 31). Rabbits are monogastric herbivores, and their intestinal structure and micro flora are different from those of pigs and cattle (26). However, there is few information about the toxic reaction of DON on rabbits and their toxicity mechanisms. Early research results illustrated that the production performance was dropped obviously after pregnant rabbits ingested 0.3 and 0.6 mg/kg b.w. DON, whereas no other toxic reactions were observed (24, 25). Another research indicated that the immune regulation of rabbits were decreased but the production performance were not changed significantly after ingested DON at 10 mg/kg feed (34). Based on relevant literature, two doses of 0.5 and 1.5 mg/kg were selected in preliminary experiments as the lower dose and the higher dose, respectively (27). The data in the current study illustrated that the intestinal structural barrier of the rabbits was damaged by DON exposure, especially in higher-dose group, and the lesions in the duodenum were more serious than those in the jejunum and ileum.

### Inflammation and DON

The small intestine is the first protective barrier against the entry of mycotoxins. Long-term DON exposure can damage the intestinal mucosa, inhibit intestinal mucosal immunity, increase the susceptibility of the body to infection, and induce symptoms such as inflammation, necrosis, and ulcers in the small intestine (35, 36). Several researches results proved that the expression of pro-inflammatory genes can be induced by DON, including cytokines, chemokines, and other genes related to inflammation (37, 38). Previous research of our laboratory revealed that DON can change the distribution and expression of pro-inflammatory factors in the small intestinal mucosa of weaned rabbits, thereby inducing inflammatory responses and intestinal damage, and these responses were related to the dose (27). The data in the current study illustrated that DON consumption increased the levels of inflammatory factors in the serum of weaned rabbits, and a trend toward dose dependency was noted.

### DON, Inflammation, and Pathway

Previous research focused on observing the toxic effects of DON on rabbits (27, 39), and this study aimed to further explored the mechanism of intestinal inflammation induced by DON. MAPKs are one of numerous signaling proteins, activating, and regulating a range of cellular activities (15). Small intestinal inflammation induced by DON may be closely associated with the activation of MAPK signaling pathways, and its toxicity is mainly achieved by activating MAPKs and changing the expression of genes associated with key physiological and immune functions in animal intestinal tissues (19, 40). It has been reported that ERK and p38, known as two important subfamily of MAPKs, play an important role in inflammatory responses induced by DON (16). ERK regulates a series of important cell biological processes, such as cell proliferation, differentiation, and apoptosis (41). And p38 plays an important role in apoptosis, cytokine production, transcriptional regulation, and cytoskeleton recognition (42). The activation of ERK and p38 can promote the production of inflammatory factors such as TNF-α, IL-1, IL-4, and so on (20, 43), as well as facilitate the expression of immune genes. DON can provoke the phosphorylation of the MAPKs, ERK, and p38, and reduce the expression of tight junction proteins by activating the MAPKs signaling pathway, thereby disrupting the function of intestinal barrier and triggering the intestinal inflammation (35, 44).

Bind-inducing process of DON was called “ribotoxic stress syndrome” activation (42). After binding with eukaryotic ribosomes, the MAPKs signaling pathway is activated by DON, and then further mediates multiple toxic effects by up-regulating inflammatory cytokines and apoptosis related genes (16). DON can trigger the gene expression of pro-inflammatory driven by p38 in cells, and these reactions require the participation of PKR and Hck, which are essential to p38 ribosome recruitment induced by DON, phosphorylation, and cause the expression of pro-inflammatory cytokine driven (39). PKR can activate the MAPKs signaling pathway in mononuclear phagocytes, and mediate a series of downstream reaction of MAPKs signaling pathway induced by DON, including the gene expression of pro-inflammatory, apoptosis, as well as modulate the expression of cytokine, such as TNF-α, IL-6, IL-12, and so on. It has been shown that PKR-mediated activation of ERK and p38 may also contribute to the up-regulation of DON-induced cytokines, and chemokines. Hck, a member of the Src family of kinases, is a non-receptor protein tyrosine kinase that is highly conserved and is a key upstream signal transduction component of the nucleotoxin stress response, which is mainly expressed in myeloid cells (45, 46). Activation of Hck may also be an important upstream signal for DON-induced MAPK phosphorylation and also for the upregulation of downstream cytokines and chemokines (47).

In this study, protein expression, relative mRNA expression, and immunoreactants of ERK1/2, p38, PCK, and Hck in different parts of the rabbit small intestine in all three groups were analyzed via Western blotting, qRT-PCR, and IHC. The results illustrated that DON intake can stimulate the expression and distribution of ERK1/2, p38, PKR, and Hck in the intestine. Furthermore, DON had the strongest effects on the expression of ERK1/2, p38, and PRK in the duodenum, whereas Hck expression was strongly altered by DON in all three intestinal segments. Meanwhile the distribution range of these factors was proportional to the intake of DON. The results of previous research in our laboratory indicated that DON can change the distribution and expression of several inflammatory cytokines in various intestinal segments of weaned rabbits (27). These trends were consistent with the findings for ERK1/2 and p38 expression in this study. However, the mechanism of DON-induced PKR and Hck expression remains unclear and requires further research.

## Data Availability Statement

The raw data supporting the conclusions of this article will be made available by the authors, without undue reservation.

## Ethics Statement

This animal study was reviewed and approved by Animal Protection Committee of Shandong Agricultural University.

## Author Contributions

PW finished the experiments. LH processed all data and wrote the draft. WY finished animal trial. QL contributed reagents tools. CW conceived the experiments and revised the paper. FL designed the study. All authors contributed to the article and approved the submitted version.

## Conflict of Interest

The authors declare that the research was conducted in the absence of any commercial or financial relationships that could be construed as a potential conflict of interest.
