# Supplementary material for: Deoxynivalenol Induces Inflammation in the Small Intestine of Weaned Rabbits by Activating Mitogen-Activated Protein Kinase Signaling
Source: Front Vet Sci. 2021 Feb 2;8:632599. doi: 10.3389/fvets.2021.632599 (PMC7884333; doi:10.3389/fvets.2021.632599)
Supplement: Supplementary file 1 [file Data_Sheet_1.PDF]

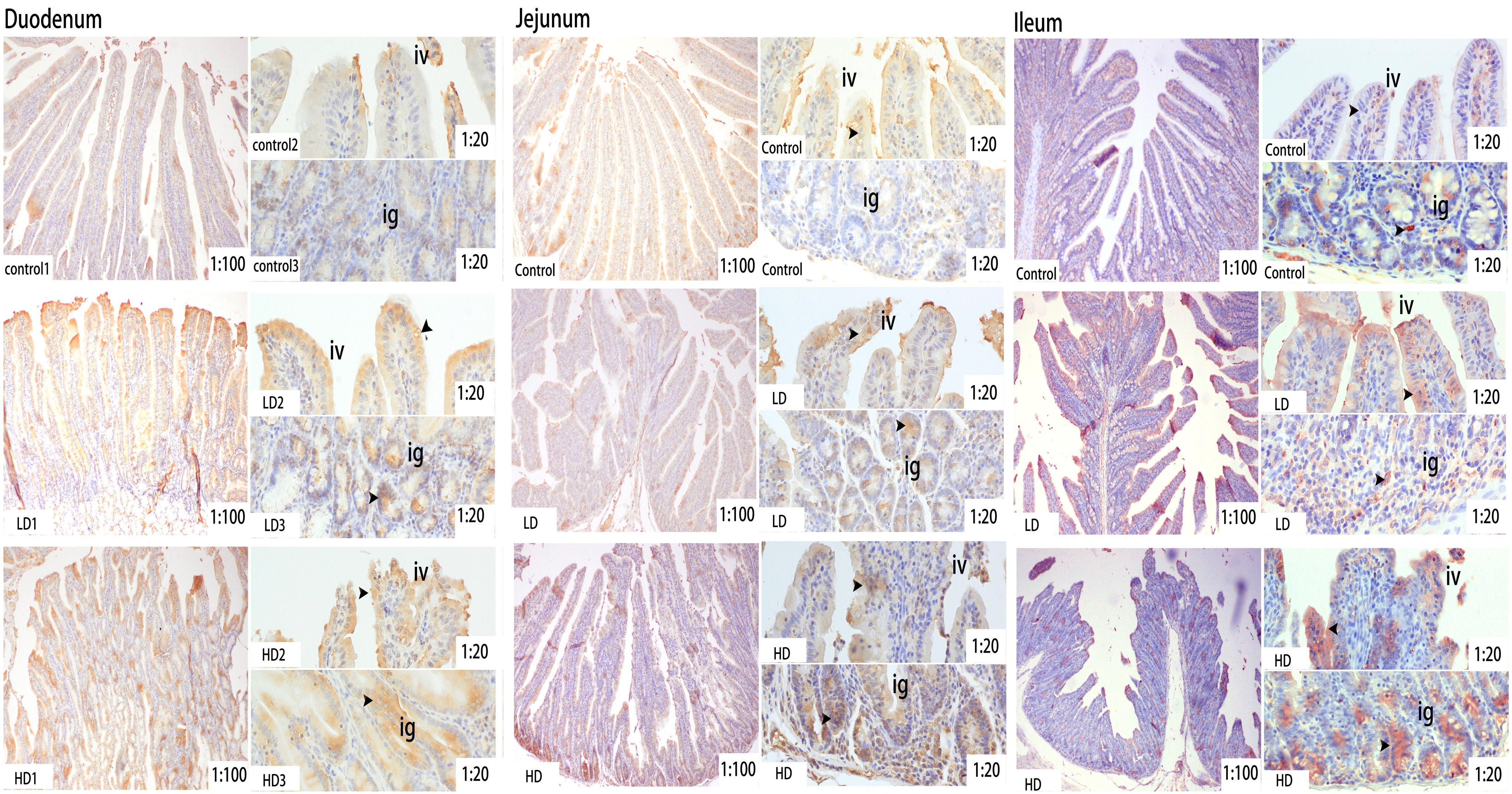

Figure S1. Representative immunohistochemistry photograph of ERK1/2. Control, LD and HD refers to the defferent treatment. iv means intestinal villus, and ig means intestinal gland. The 1:100 and 1:20 represent the magnification of electron microscopy is 10 × and 40 × respectively. The brown positive reactants was emphasized by black arrow.

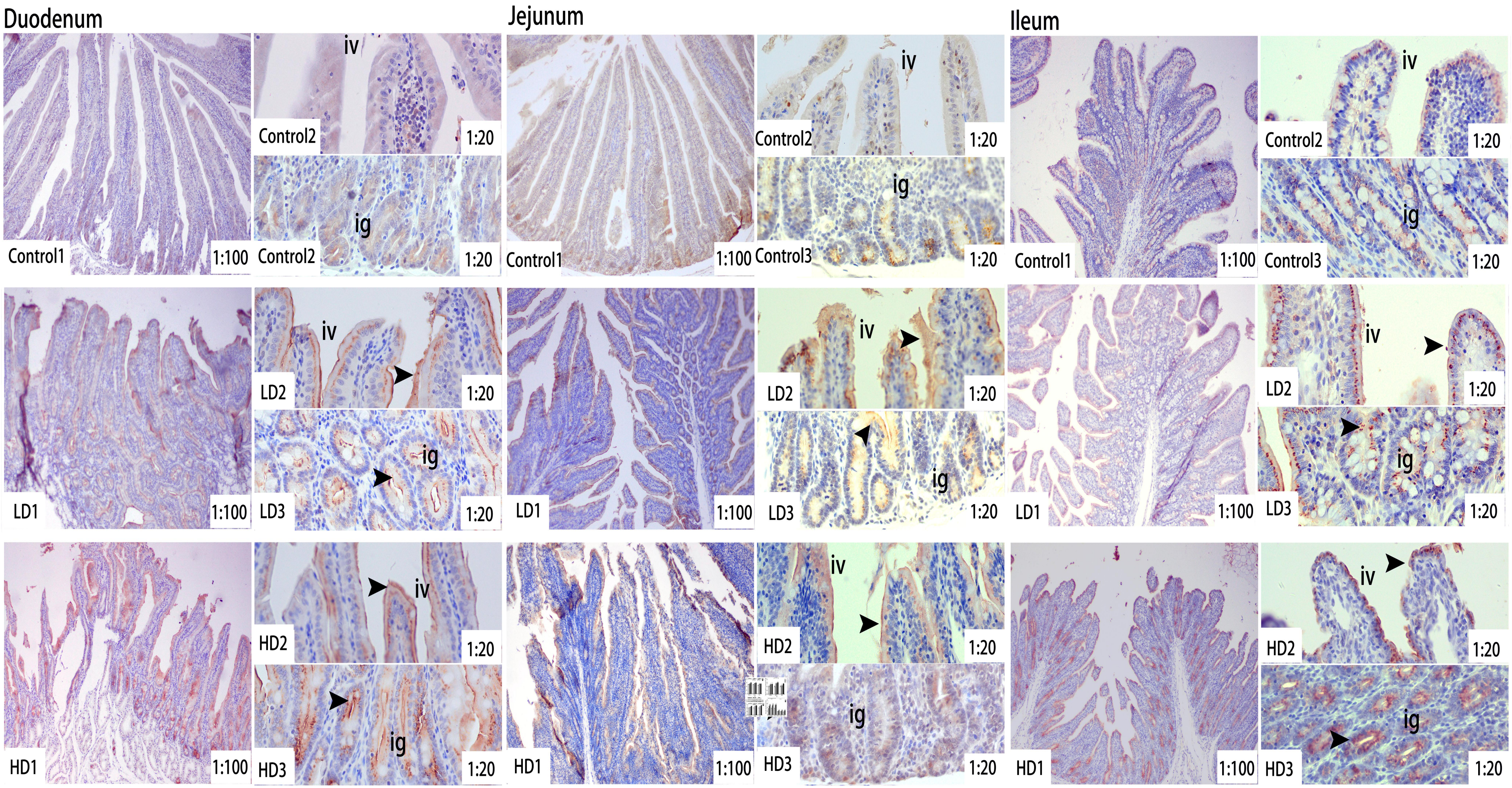

Figure S2. Representative immunohistochemistry photograph of p38. Control, LD and HD refers to the defferent treatment. iv means intestinal villus, and ig means intestinal gland. The 1:100 and 1:20 represent the magnification of electron microscopy is 10 × and 40 × respectively. The brown positive reactants was emphasized by black arrow.

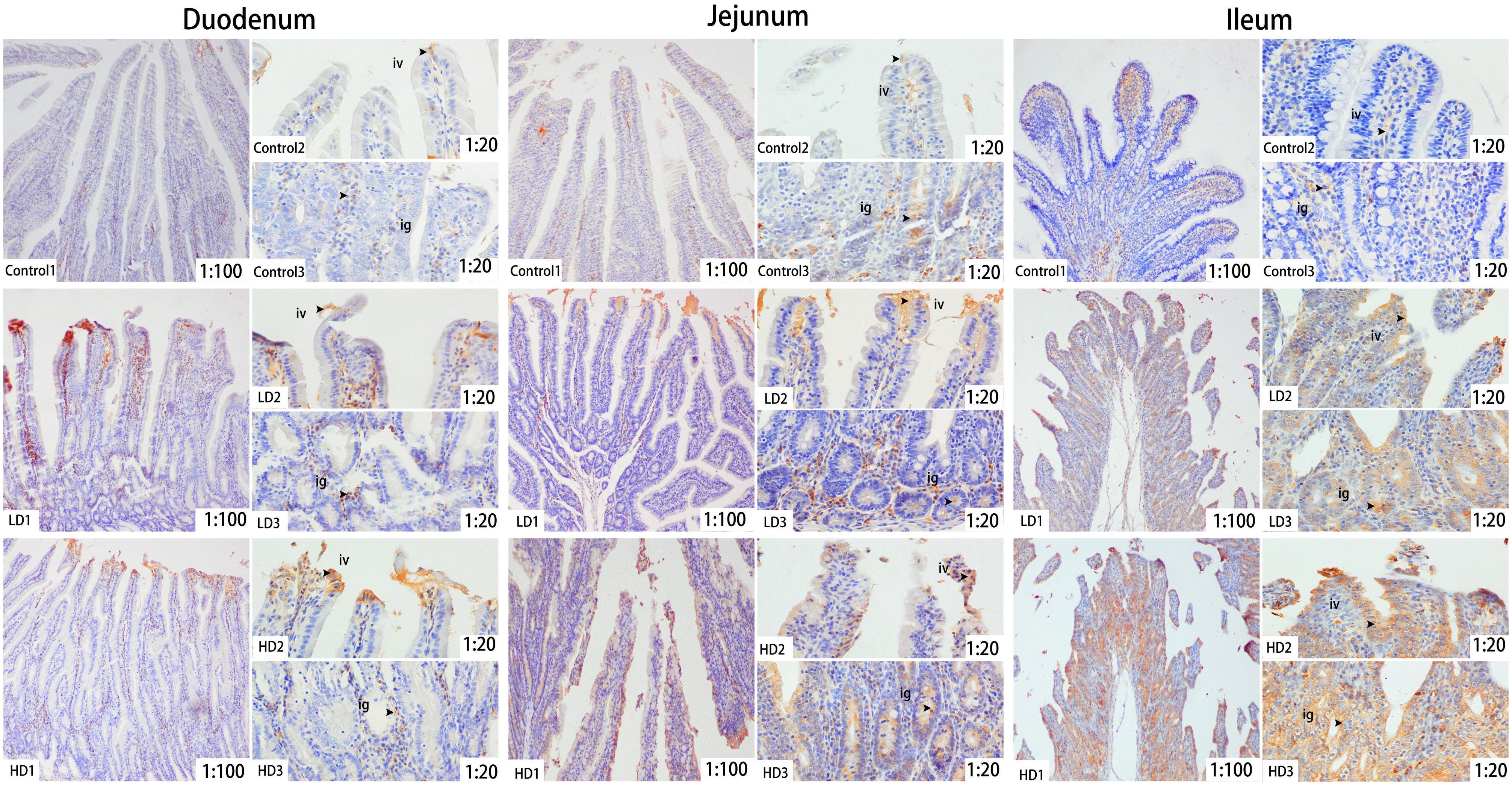

Figure S3. Representative immunohistochemistry photograph of PKR. Control, LD and HD refers to the defferent treatment. iv means intestinal villus, and ig means intestinal gland. The 1:100 and 1:20 represent the magnification of electron microscopy is 10 × and 40 × respectively. The brown positive reactants was emphasized by black arrow.

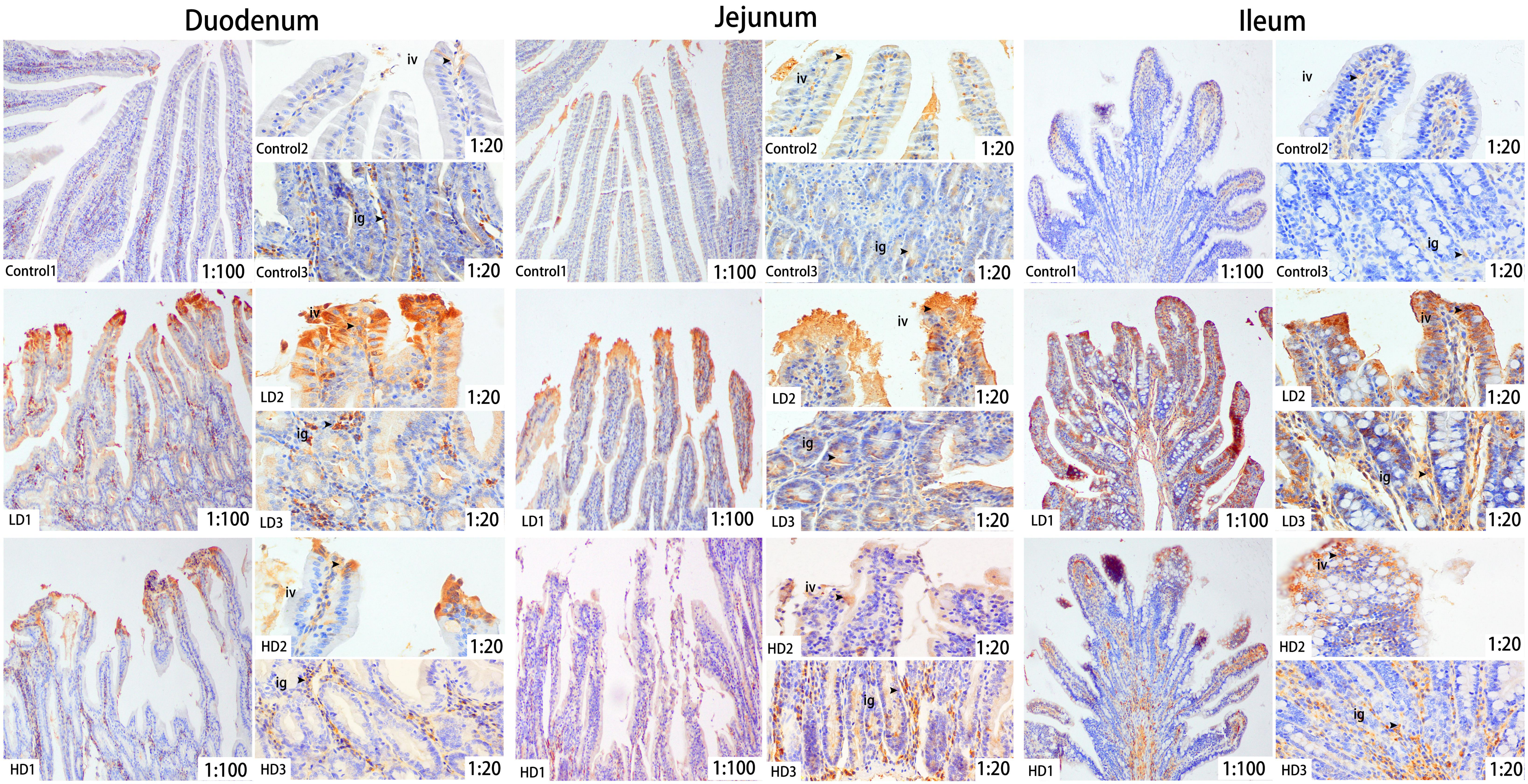

Figure S4. Representative immunohistochemistry photograph of Hck. Control, LD and HD refers to the defferent treatment. iv means intestinal villus, and ig means intestinal gland. The 1:100 and 1:20 represent the magnification of electron microscopy is 10 × and 40 × respectively. The brown positive reactants was emphasized by black arrow.
